# Comprehensive Optimization of Emergency Evacuation Route and Departure Time under Traffic Control

**DOI:** 10.1155/2014/870892

**Published:** 2014-04-13

**Authors:** Guo Li, Ying Zhou, Mengqi Liu

**Affiliations:** ^1^School of Management and Economics, Beijing Institute of Technology, Beijing 100081, China; ^2^Business School, Hunan University, Changsha 410082, China

## Abstract

With the frequent occurrence of major emergencies, emergency management gets high attention from all around the world. This paper investigates the comprehensive optimization of major emergency evacuation route and departure time, in which case the evacuation propagation mechanism is considered under traffic control. Given the practical assumptions, we first establish a comprehensive optimization model based on the simulation of evacuation route and departure time. Furthermore, we explore the reasonable description method of evacuation traffic flow propagation under traffic control, including the establishment of traffic flow propagation model and the design of the simulation mudule that can simulate the evacuation traffic flow. Finally, we propose a heuristic algorithm for the optimization of this comprehensive model. In case analysis, we take some areas in Beijing as the evaluation sources to verify the reliability of our model. A series of constructive suggestions for Beijing's emergency evacuation are proposed, which can be applied to the actual situation under traffic control.

## 1. Introduction

In recent years we have witnessed a significant increase in the incidence of major natural disasters. For example, disastrous emergencies such as SARS, Indonesian tsunami, and Japan earthquake occur frequently all around the world. On a global scale, the losses caused by disasters such as super tornadoes in the southern United States, floods in Queensland, and the earthquakes that hit Australia and New Zealand reached the level which only happened once in ten years or even decades. Also, the Japanese earthquake and the following nuclear leak as a whole became the second largest catastrophic event in the history (measured by the insurance loss). The total economic losses caused by disasters in the first half of 2011 were close to $278 billion, which were second only to the losses in 2005 (the most serious year in the history). For China, many major natural disasters such as severe snow and ice in northern China, the 5.8 magnitude earthquake in Yunnan, Yingjiang, the rainstorm and flood in southern China, and coastal typhoon occurred one after another, and all of that mentioned above had great impacts on the development of economy and society, people's life, and property safety. According to the statistics, in 2011, due to natural disasters, 430 million people were affected, 1126 people were killed (including the missing 112 people), 9.394 million people were evacuated, and the direct economic losses hit 309.64 billion RMB (excluding the data in Hong Kong, Macao, and Taiwan).

With the further acceleration of urbanization, population becomes more concentrated. Thus, it is important to figure out how to respond to the emergencies timely and effectively. Emergency evacuation, as a main means of rapid evacuation and reducing losses to the minimum in times of crisis, has been on the front burner in the emergency management.

According to the scale, evacuation issues can be divided into small scale evacuation and long-distance regional evacuation. Small scale evacuation generally refers to the evacuation aiming at swift and violent emergencies that only affect small space, such as explosion within a finite range, house collapse, and fire breaking out in shopping malls. Usually, evacuation of this kind is mainly dealt with walking evacuation artificially, not requiring means of transport. While the long-distance regional evacuation generally needs vehicles for transport, and it is often commanded by the government or related departments with mandatory measures. Therefore, the evacuation problems discussed in this paper all belong to long-distance regional and mandatory evacuation.

The rest of our paper is organized as follows.  Section 2 reviews the related literature.  Section 3 establishes a comprehensive optimization model based on the simulation of evacuation route and departure time.  Section 4 develops the reasonable description method of evacuation traffic flow propagation under traffic control.  Section 5 provides a heuristic algorithm for the optimization of this comprehensive model. Case analysis and tests are reported in Section 6. The concluding remarks are given in Section 7.

## 2. Literature Review

Our paper belongs to the vast literature that investigates the optimization of emergency evacuation route and departure time. In the prior literature, many scholars have studied this issue from different perspectives, for example, emergency evacuation model [1–8], evacuation simulation [9–13], optimal route finding [14, 15], and the algorithm [16–18]. Among them, Antoine and Erol [1] propose an approach that offers fast estimates based on graph models and probability models. They show that graph models can offer insight into the critical areas in an emergency evacuation and that analytical models based on queuing theory can provide useful estimates for the optimization of evacuation time and route. Chen and Xiao [4] establish an optimal objective based on the shortest emergency time and acquire the optimal solution using the Pontryagin minimum principle. The evacuation route construction algorithm and traffic flow assignment algorithm in each junction are employed to deliver the traffic flow in the evacuation area to a safe region rapidly and safely. The idea of feedback is introduced in the execution using real-time information to adjust and update the evacuation plan. Hasby and Khodra [14] discuss Twitter-based traffic information extraction and its usage as heuristic in optimal route finding. Their system is divided into two modules, extraction information and route finding, and displays a map with marked route based on traffic information extracted from Twitter.

Lu and Betsy [17] present a heuristic algorithm, namely, capacity constrained route planner (CCRP), which models capacity as a time series and uses a capacity constrained routing approach to incorporate route capacity constraints. The CCRP algorithm produces high quality solutions and significantly reduces the computational cost compared to linear programming approach. Papinigis et al. [19] present a calculation method for people evacuation based on the physical characteristics of people stream (density, intensity, and movement speed). The time required to evacuate people from the building is determined in the numerical illustration of the method application. Furthermore, they make the comparison between simple calculation method and modeling with FDS + Evac software.

In addition, on the evacuation problem of nuclear power plant surrounding areas, Dunn and Newton [20] treat evacuation traffic flow and evacuation time as quantitative, explore how to evacuate people as far as possible within the prescribed time, and consider the evacuation as maximum-flow problem to find the optimal evacuation route. On the evacuation problem of earthquake, Yamada [21] describes route optimization of neighborhoods getting to their respective shelters as shortest route problem and the minimum cost flow problem. By finding the shortest route and minimum cost flow, evacuation planning is obtained. Campos et al. [22] firstly analyze how to reduce the conflict between the evacuation traffic flow and increase the traffic capacity of evacuation route, then treat the ratio of the traffic capacity of route and travel time as a measurable index of traffic performance, finally put forward a heuristic algorithm to find the best route between OD points. Satoko et al. [23] describe the optimization of evacuation route and departure time as the fastest flow problem under the premise that the traffic starting from the same point can only depart for a designated ending point, and they provide an algorithm to find the fastest flow in the tree network.

Our paper differs from the above literature in the following aspects. First, traffic flow propagation in emergency evaluation should be considered as it affects the evaluation measures. Besides, traffic control and regulations in turn will have great effects on traffic flow propagation. As a result, our paper investigates the optimization of emergency evacuation route and departure time considering the interaction between traffic control and traffic flow propagation. Moreover, we not only establish a simulation model but also design a similar heuristic algorithm. At last, we choose some areas in Beijing to verify the reliability of the model.

## 3. Comprehensive Optimization Model

### 3.1. Assumptions and Parameter Definitions

A comprehensive optimization model is established based on the simulation of evacuation route and departure time and its premises and assumptions are as follows.

1. The traffic volume (or the number of vehicles) at evacuation source that needs to be evacuated to the ending point is known.
2. The traffic volume is 0 at the initial time in the road network, regardless of the traffic that has already existed in the network.
3. There exist only evacuation traffic flow and rescue traffic flow in the network during the evacuation period, and evacuation traffic flow is effectively separate from the rescue traffic flow under traffic control.

Nodes include the intersection points, evacuation sources, and ending points. Arcs mean the distance between adjacent intersections or the distance between the intersection and evacuation source or ending point. By removing the arcs with one-way and prohibition sections and changing the number of lanes and traffic capacity of the arcs with the sections that have reverse traffic, the evacuation network can be abstracted into a directed network *G* = {*V*, *A*}, where *V* is the set of nodes and *A* is the set of arcs. Assume that the evacuation happens in [0, *T*], which means that the starting time of the evacuation is 0 and the ending time is *T*. Divide [0, *T*] into *K* parts, each of which is Δ, so *T* = *K*Δ. Symbols and variables in this paper are defined as follows.

 
*M*: set of evacuation source nodes in the road network.
 
*N*: set of evacuation ending nodes in the road network.
 
*m*: evacuation source node, where *m* ∈ *M*.
 
*n*: evacuation ending node, where *n* ∈ *N*.
 
*a*: section in the evacuation network, where *a* ∈ *A*.
 
*p*: intersection in the evacuation network, where *p* ∈ *V*.
 
*R*_*mn*_: set of routes between the evacuation source *m* and the ending node *n*.
 
*l*: serial-number of the period in which vehicles depart from the evacuation source, where *l* = 1,2,…, *K*.
 
*l*_*s*_, *l*_*e*_: starting (ending) point of period *l*, *l*_*s*_ = (*l* − 1)Δ, *l*_*e*_ = *l*_*s*_ + Δ.
 
*k*: serial-number of the period in which vehicles run in the road network, *k* = 1,2,…, *K*.
 
*k*_*s*_, *k*_*e*_: starting (ending) point of period *k*, *k*_*s*_ = (*k* − 1)Δ, *k*_*e*_ = *k*_*s*_ + Δ.
 
*D*_*mn*_: total evacuation demand (or the number of vehicles) between *m* and *n*.
 
*F*_*mnr*_^*l*^: number of vehicles that depart from *m* to *n* along route *r* in period *l*, where *r* ∈ *R*_*mn*_.
 
*F*: set of *F*_*mnr*_^*l*^, for all *m*, *n*, *r*, *l*.
 
*p*_*a*_^*k*^: number of vehicles running on section *a* at the starting point of period *k*.
 
*x*_*a*_^*k*^, *y*_*a*_^*k*^: number of vehicles that enter (leave) section *a* in period *k*.
 
*x*_*mnr*_^*lka*^, *y*_*mnr*_^*lka*^: number of vehicles that enter (leave) section *a* in period *k* among those that depart from *m* to *n* along route *r* in period *l*.
 
*t*_*mnr*_^*l*^(*F*): average running time spent by vehicles that depart from *m* to *n* along route *r* in period *l* under the traffic condition determined by *F*.
 
*A*(*p*), *B*(*p*): set of sections, and of which the starting (ending) nodes are *p*.
 
*δ*_*mnr*_^*lka*^: value is 0 or 1. If vehicles that depart from *m* to *n* along route *r* in period *l* enter section *a* in period *k*, then 1 is taken; otherwise 0 is taken.

### 3.2. Model Formulation

The objective of an evacuation organization planning is to get the shortest total evacuation time with reasonable arrangement for departure time and evacuation route, which can be described as

(1) $$\begin{array}{c} \mathrm{Min}T. \end{array}$$

Though formula (1) is intuitive and simple, it is unable to establish contact with decision variables. Therefore, the following formula can be expanded from formula (1) as the objective function:

(2) $$\begin{array}{c} \mathrm{Min}Z\left(F\right)=\underset{m\in M\;}{\sum }\underset{n\in N\;}{\sum }\underset{r\in R_{mn}\;}{\sum }\overset{K}{\underset{l=1}{\sum }}F_{mnr}^{l}\left[\left(l_{s}+\alpha \Delta \right)+t_{mnr}^{l}\left(F\right)\right], \end{array}$$

where *l*_*s*_ + *α*Δ means the average waiting time spent by vehicles that depart in period *l* at evacuation source, and 0 ≤ *α* ≤ 1.

Constraints of the model are as follows:

(3) $$\begin{array}{c} \underset{r\in R_{mn}\;}{\sum }\underset{1\leq l\leq K}{\sum }F_{mnr}^{l}=D_{mn}\;\forall m,n, \end{array}$$

(4) $$\begin{array}{c} p_{a}\left(0\right)=0\;\forall a, \end{array}$$

(5) $$\begin{array}{c} p_{a}^{k}=p_{a}^{k-1}+x_{a}^{k}-y_{a}^{k}\;\forall k,a, \end{array}$$

(6) ∑b∈B(p1)xbk=∑c∈A(p2)yck

(7) $$\begin{array}{c} x_{a}^{k}=\underset{m\in M\;}{\sum }\underset{n\in N\;}{\sum }\underset{r\in R_{mn}\;}{\sum }\underset{1\leq l\leq K}{\sum }x_{mnr}^{lka}\;\forall k,a, \end{array}$$

(8) $$\begin{array}{c} y_{a}^{k}=\underset{m\in M\;}{\sum }\underset{n\in N\;}{\sum }\underset{r\in R_{mn}\;}{\sum }\underset{1\leq l\leq K}{\sum }y_{mnr}^{lka}\;\forall k,a, \end{array}$$

(9) $$\begin{array}{c} \left(\begin{array}{c} x_{mnr}^{lka}\\ y_{mnr}^{lka}\\ \delta_{mnr}^{lka} \end{array}\right)=g\left(F_{mnr}^{l}:\forall m,n,r,l\right)\;\forall m,n,r,k,l,a, \end{array}$$

(10) $$\begin{array}{c} t_{mnr}^{l}\left(F\right)=\underset{a\in A\;}{\sum }\overset{K}{\underset{k=1}{\sum }}\delta_{mnr}^{lka}\cdot \Delta \;\forall m,n,r,l, \end{array}$$

where all variables are greater than or equal to 0.

Formula (3) is the conservation constraint of total evacuation demand, which means that the total number of vehicles that depart along all dynamic routes between the evacuation OD is equal to the total evacuation demand. Formula (4) gives the initial condition of the road network, which means there is no vehicle at the initial time. Formula (5) is the state equation of the section, which describes the dynamic changes of the number of vehicles in the section. Formula (6) is the conservation constraint of the node, which means vehicles cannot stop at the node, and in each period the total number of vehicles arriving at a node is equal to the total number of vehicles leaving this node. Formulas (7) and (8) give the calculation method of outflow and inflow of the dynamic section. Formula (9) represents a constraint of traffic flow propagation, in which the function *g*() describes the relationship between the state variables based on section and the decision variables based on route. The detailed description will be illustrated by simulation method in Section 4. Formula (10) is used to calculate the running time on the route and it also shows the relationship between decision variables and objective function.

## 4. Traffic Flow Propagation Mechanism

### 4.1. Model Description and Parameter Definitions

In this section, we will explore the traffic flow propagation mechanism. In order to reflect the bottleneck effect which is exerted by control measures at the intersection in the process of traffic flow propagation, we need to, respectively, describe the dynamic outflow of each entrance lane. In addition, regardless of the microbehaviors of vehicles in the running process, such as lane changing and overtaking, it can be argued that traffic flow propagation in a section (or in the running process) is subject to the same law in an average sense.

Thus, this paper establishes the comprehensive model that includes both running time function and shunted outflow function to describe the traffic flow propagation in a section and between two sections at the intersection. The model is divided into two parts in theory: the running part and the queuing part. Running time function describes the traffic flow propagation in the running part, and shunted outflow function describes the traffic flow propagation in the queuing part. Shunted outflow function divides the queuing part at the end of the section into multiple queues, and the traffic flow propagation between sections can be described by the dynamic outflow rate of each queue.

We use *G* = {*V*, *A*} to stand for the evacuation network and divide the period [0, *T*] into *M* intervals. The length of each interval is *σ*  (*σ* < Δ), which is small enough to guarantee that the inflow and outflow rates of the section remain unchanged in each interval. In order to distinguish *σ* and Δ, here we assume time *t* is a small period whose length is *σ*. For the sake of simplicity, we convert all time variables to integer times of *σ*. Symbols and variables in this model are defined as follows.

 
*t*: current (discretization) time, *t* = 1,2,…, *M*.
 
*f*_*mnr*_(*t*): departure (or outflow) rate of route *r* between *m* and *n* at time *t*, where *r* ∈ *R*_*mn*_.
 
*xr*_*mnr*_^*a*^(*t*), *yr*_*mnr*_^*a*^(*t*): flow rate while vehicles that depart from *m* to *n* along route *r* are entering (leaving) the running part of section *a* at time *t*.
  
*xq*_*mnr*_^*aa*^+^^(*t*), *yq*_*mnr*_^*aa*^+^^(*t*): flow rate while vehicles that depart from *m* to *n* along route *r* are entering (leaving) the turning queue from section *a* to section *a* ^+^ (the downstream section of this turning is section *a* ^+^) at time *t*.
 
*nr*_*mnr*_^*a*^(*t*): number of vehicles that depart from *m* to *n* along route *r* in the running part of section *a* at time *t*.
 
*nq*_*mnr*_^*aa*^+^^(*t*): number of vehicles that depart from *m* to *n* along route *r* among those that are in the turning queue from section *a* to section *a* ^+^ at time *t*.
 
*nr*_*a*_(*t*): number of vehicles that are in the running part of section *a* at time *t*.
 
*nq*_*aa*^+^_(*t*): number of vehicles that are in the turning queue from section *a* to section *a* ^+^ at time *t*.
 
*nq*_*a*_(*t*): number of vehicles that are in the queuing part of section *a* at time *t*, and it is equal to the sum of the number of vehicles in all turning queues at that time.
 
*xq*_*aa*^+^_(*t*), *yq*_*aa*^+^_(*t*): inflow rate (outflow rate) of the turning queue from section *a* to section *a* ^+^ at time *t*.
 
*xq*_*a*_(*t*),*yq*_*a*_(*t*): total inflow (outflow) rate of section *a*, and it is equal to the sum of the inflow (outflow) rates of all turning queues at time *t*.
 
*tr*_*a*_(*t*): running time of vehicles that enter section *a* at time *t*.
 
*tq*_*aa*^+^_(*t*): waiting time (or the delay) of vehicles that enter the turning queue at time *t* after leaving the running part of section *a*.
 
*h*_*a*_: maximum number of vehicles in section *a* (associated with the geometric conditions and jam density of the section).
 
*s*_*aa*^+^_: maximum outflow rate of the turning queue from section *a* to section *a* ^+^ (determined by the capacity of the entrance lane in this turning direction).
 
*s*_*a*_: maximum inflow rate of section *a* (determined by the capacity of this section).
 
*a*_*h*_, *a*_*t*_: terminal node (beginning node) of section *a*.

### 4.2. Running Part

Traffic flow propagation in the running part is described by running time function, namely, the transformation of basic speed formula:

(11) $$\begin{array}{c} T^{\prime }=\frac{L}{V}, \end{array}$$

where *T*′ is the running time, *L* is the length of the section, and *V* is the running speed. Due to the existence of intersections, the actual running distance is smaller than the length of the section. But compared to the running distance, the distance at the intersection is small enough to be ignored. Therefore, here, the running distance can be the substitute of the length of the section. The limit of the running speed is the maximum speed, which varies in different sections.

In section *a*, the running time can be calculated:

(12) $$\begin{array}{c} tr_{a}\left(t\right)=\frac{L_{a}}{V_{a}}. \end{array}$$

The aim of the description of traffic flow propagation in the running part is to get its outflow rate based on the inflow rate, running time and number of vehicles in the section, as shown in the following formulas:

(13) yrmnra(t+tra(t))=xrmnra(t)l+tra(t)−tra(t−1),∀m,n,t,a,  ∀r∈Rmn,

(14) yrmnra(i)=yrmnra(j)+(i−j)yrmnra(j′)−yrmnra(j)j′−j,∀m,n,a, ∀r∈Rmn,

where *i* is an integer, *j* = *t* + *tr*_*a*_(*t*), and *j*′ = *t* + 1 + *tr*_*a*_(*t* + 1).

Formula (14) is the linear approximation of the outflow rates that are not at integer time.

The state of the running part, namely, the number of vehicles, can be updated by the following formulas:

(15) $$\begin{array}{c} nr_{mnr}^{a}\left(t+1\right)=nr_{mnr}^{a}\left(t\right)+xr_{mnr}^{a}\left(t\right)-yr_{mnr}^{a}\left(t\right) \end{array}$$

(16) $$\begin{array}{c} nr_{a}\left(t\right)=\underset{m\in M\;}{\sum }\underset{n\in N\;}{\sum }\underset{\begin{array}{c} r:\in R_{mn}\\ a\in r \end{array}}{\sum }nr_{mnr}^{a}\left(t\right),\;\forall a,t. \end{array}$$

### 4.3. Queuing Part

The purpose of the description of traffic flow propagation in the queuing part is mainly to get the actual outflow rate based on the outflow demand and downstream supply. The outflow demand of the queue is determined by the inflow rate, number of vehicles in the queue and biggest outflow rate of the entrance lane at that time, and the downstream supply is determined by the space capacity, maximum inflow rate and total outflow rate at that time.

The inflow rate of each turning queue is equal to the outflow rate in the corresponding turning direction in the running part, as shown in following formulas:

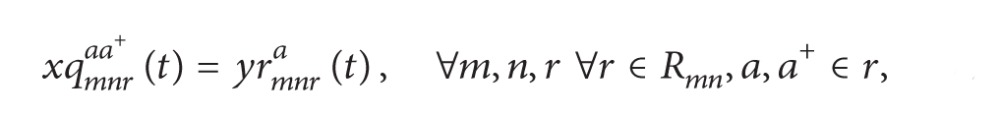
(17)

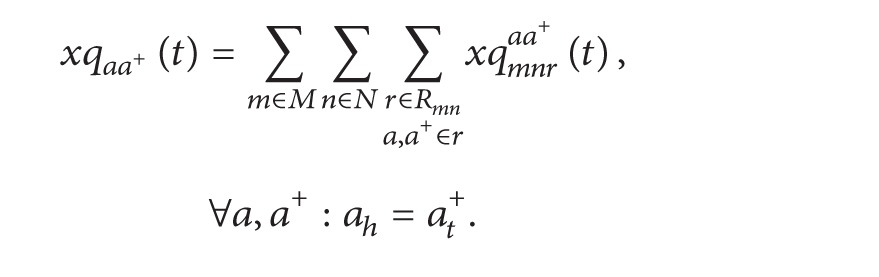
(18)


The outflow rate in the queuing part can be calculated by the following formula based on its own outflow demand and downstream supply:

(19) $$\begin{array}{c} {yq}_{aa^{+}}\left(t\right)=\min\left[M_{aa^{+}}\left(t\right),N_{aa^{+}}\left(t\right)\right]\;\forall a,a^{+}:a_{h}=a_{t}^{+}. \end{array}$$

*N*_*aa*+_(*t*) and *M*_*aa*^+^_(*t*), respectively, represent the outflow demand in the turning queue from section *a* to section *a* ^+^ and the supply capacity in section *a* ^+^ that can be assigned to section *a* at time *t*. *N*_*aa*^+^_(*t*) can be obtained by formula (20), and *M*_*aa*^+^_(*t*) can be calculated by formulas (21) and (22).

Consider the following:

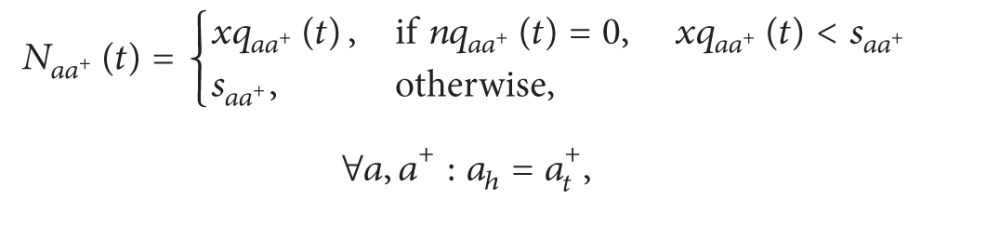
(20)

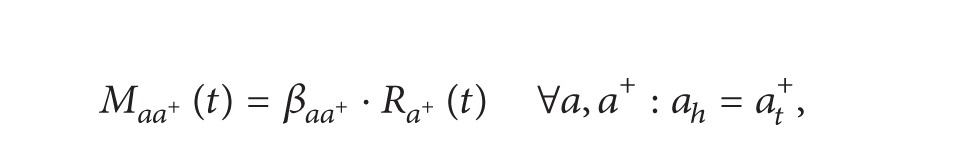
(21)

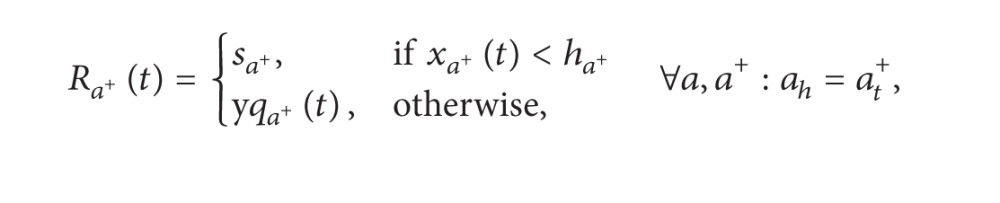
(22)
where *β*_*aa*^+^_ is supply allocation coefficient, the value of which is related to the proportion of lanes between upstream and downstream sections and the control measures at the intersection, and it is regarded as a fixed constant. The total outflow rate of the section can be measured by the following formula after calculating the outflow rate in each turning direction in the queuing part:

(23) $$\begin{array}{c} yq_{a}\left(t\right)=\underset{a^{+}:a_{h}=a_{t}^{+}}{\sum }yq_{aa^{+}}\left(t\right)\;\forall a. \end{array}$$

Assume that vehicles in each queue on entrance lane are evenly mixed together, and the outflow rate of the queue on specific route can be calculated by the following formula:

(24) $$\begin{array}{c} yq_{mnr}^{aa^{+}}\left(t\right)=\{\begin{array}{cc} \frac{nq_{mnr}^{aa^{+}}}{nq_{aa^{+}}\left(t\right)}\cdot yq_{aa^{+}}\left(t\right), & \text{if}\;\;nq_{aa^{+}}\left(t\right)>0\\ \frac{xq_{mnr}^{aa^{+}}}{xq_{aa^{+}}}\cdot yq_{aa^{+}}\left(t\right), & \text{otherwise}, \end{array}\\ \;\;\;\;\;\forall m,n,r,\;\forall r\in R_{mn},a,a^{+}\in r. \end{array}$$

The number of vehicles in each queue can be updated by the following formulas:

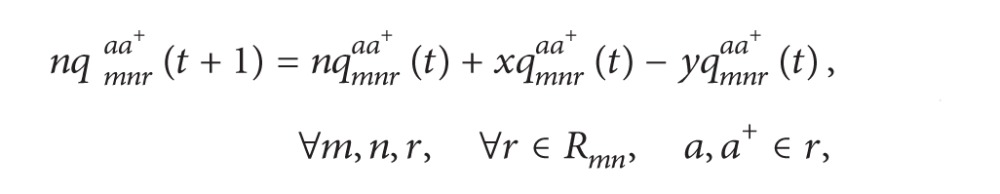
(25)

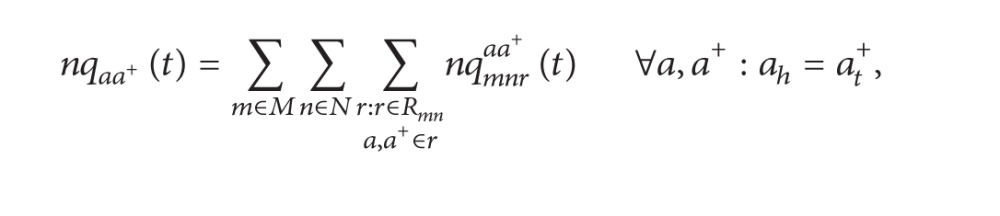
(26)

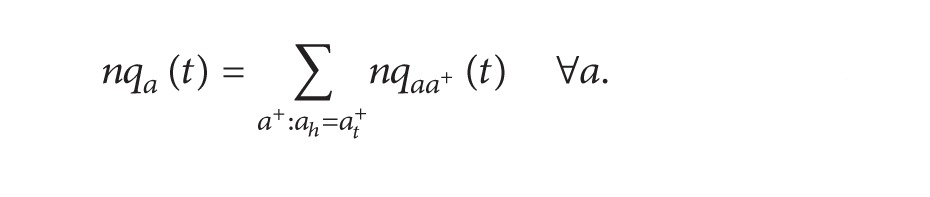
(27)


### 4.4. Simulation of Evacuation Traffic Flow

This part is about the simulation process of traffic flow entering and running in the road network with the given evacuation route and departure time. The simulation can transform the state description of the road network based on route into that based on section, and finally contribute to the optimization and evaluation of evacuation organization planning.

According to the optimization model of evacuation route and departure time, simulation inputs are the departure time and running routes of the vehicles (or in each decision period, the number of vehicles that depart along different routes). Assume that vehicles evenly depart from the evacuation source in each decision period. For example, there are 50 vehicles leaving the evacuation source along certain route in 10 minutes, then it can be described as that these 50 vehicles depart evenly in 10 minutes with a flow rate of 5 vehicle/min. The departure rate of the dynamic route can be measured by the following formula:

(28) $$\begin{array}{c} {f_{mnr}^{l}}_{\;}\left(t\right)=\frac{F_{mnr}^{l}}{\Delta }\;l_{s\;}\leq t\cdot \sigma \leq l_{e},\forall l. \end{array}$$

In addition, for the given evacuation organization planning *f*_*mnr*_^*l*^(*t*) is included in the following formula:

(29) $$\begin{array}{c} xr_{mnr}^{a}\left(t\right)=\{\begin{array}{cc} f_{mnr}^{l}\left(t\right), & \text{if}\;a\;\text{is}\;\text{the}\;\text{first}\;\text{section}\;\mathrm{on}\;\text{route}\;r\\ yq_{mnr}^{(a-r)a}\left(t\right), & \text{otherwise}, \end{array}\\ \;\;\;\;\;\;\;\;\;\;\;\;\;\;\forall m,n,a,\;\forall r\in R_{mn}, \end{array}$$

where *a*-*r* is the section in front of section *a* on route *r*.

The steps of the simulation are as follows.

**Step 1**

Let *t* = 1,  *xr*_*mnr*_^*a*^(*t*) = 0, and *nq*_*mnr*_^*aa*+^(*t*) = 0, for all *m*, *n*, *t*, *r*, for all *a*,  *a*_*t*_^+^ = *a*_*h*_.

**Step 2**

Calculation and derivation in the running part.

1. According to the given inputs, *xr*_*mnr*_^*a*^(*t*) can be measured by formulas (28)-(29).
2. Calculate *nr*_*a*_(*t*) by formula (16) and *nq*_*a*_(*t*) by formulas (26) and (27).
3. Calculate *tr*_*a*_(*t*) for all *t*, *a* by formula (12).
4. Calculate *yr*_*mnr*_^*a*^(*t* + *tr*_*a*_(*t*)) and *yr*_*mnr*_^*a*^(*j*), for all *m*, *n*, *r*, *t*, *a*, *j* ∈ [*t* + *tr*_*a*_(*t*), *t* + 1 + *tr*_*a*_(*t* + 1)] and *j* is an integer by formulas (13)-(14).
5. Calculate *nr*_*mnr*_^*a*^(*t*) for all *m*, *n*, *r*, *t*, *a* by formula (15).

**Step 3**

Calculation and derivation in the queuing part.

1. Calculate *xq*_*mnr*_^*aa*^+^^(*t*),for all *m*, *n*, *r*, *t*, for all *a* : *a*_*t*_^+^ = *a*_*h*_ by formula (17).
2. Calculate *xq*_*aa*^+^_(*t*), *nq*_*aa*^+^_(*t*), for all *t*, *a*, *a* ^+^ : *a*_*t*_^+^ = *a*_*h*_ by formulas (18) and (26).
3. Calculate *yq*_*aa*^+^_(t), *yq*_*a*_(*t*), for all *t*, *a*, *a* ^+^ : *a*_t_^+^ = *a*_*h*_ by formulas (19)–(23).
4. Calculate *yq*_*mnr*_^*aa*^+^^(*t*) and *nq*_*mnr*_^*aa*^+^^(*t*), for all *m*, *n*, *r*, *t*, for all *a* : *a*_*t*_^+^ = *a*_*h*_ by formulas (24) and (25).

**Step 4**

If *t* = *M*, then stop; otherwise let *t* = *t* + 1, and go back to Step 2.

## 5. Model Solution

### 5.1. Optimal Conditions Analysis

Among all the constraints from formulas (2) to (10) shown in the model, what really affect the decision variables are the conservation constraint of total evacuation demand in formula (3) and the nonnegative constraints on the decision variables (or *F*_*mnr*_^*l*^ ≥ 0). Actually, the objective of our model is to find the minimum with a series of equation constraints. Thus we can establish the Lagrange function based on formulas (2) and (3):

(30) $$\begin{array}{c} L\left(F,\theta \right)=Z\left(F\right)+\underset{m}{\sum }\underset{n}{\sum }\theta_{mn}\left(D_{mn}-\underset{r}{\sum }\underset{l}{\sum }F_{mnr}^{l}\right), \end{array}$$

where *θ* stands for Lagrange multiplier vector, and *θ*_*mn*_ represents two-dimensional Lagrange multiplier.

According to the Kuhn-Tucker conditions, the Lagrange function above must meet the following conditions at the extreme point:

(31) $$\begin{array}{c} F_{mnr}^{l}\frac{\partial L\left(F,\theta \right)}{\partial F_{mnr}^{l}}=0,\;\;\frac{\partial L\left(F,\theta \right)}{\partial F_{mnr}^{l}}\geq 0\;\forall m,n,r,l, \end{array}$$

(32) $$\begin{array}{c} \frac{\partial L\left(F,\theta \right)}{\partial \theta_{mn}}=0\;\forall m,n. \end{array}$$

For certain *F*_*ijw*_^*s*^, where *i* = *m*, *j* = *n*, *w* = *r*, *s* = *l*, the partial derivative of the Lagrange function can be calculated as follows:

(33) ∂L(F,θ)∂Fijws=∂Z(F)∂Fijws +∂∂Fijws[∑m∑nθmn(Dmn−∑r∑lFmnrl)]∀i,j,w,s.

There is no relationship between *F*_*ijw*_^*s*^ and *θ*_*mn*_, also between *F*_*ijw*_^*s*^ and *D*_*mn*_.

What is more,

(34) $$\begin{array}{c} \frac{\partial F_{mnr}^{l}}{\partial F_{ijw}^{s}}=\{\begin{array}{cc} 1, & \text{if}\;\;m=i,\;\;n=j,\;\;r=w,\;\;l=s\\ 0, & \text{others}. \end{array} \end{array}$$

Therefore, we can get

(35) $$\begin{array}{c} \frac{\partial }{\partial F_{ijw}^{s}}\left[\underset{m}{\sum }\underset{n}{\sum }\theta_{mn}\left(D_{mn}-\underset{r}{\sum }\underset{l}{\sum }F_{mnr}^{l}\right)\right]=-\theta_{ij}. \end{array}$$

Let

(36) $$\begin{array}{c} \beta_{ijw}^{s}\left(F\right)=\frac{\partial Z\left(F\right)}{\partial F_{ijw}^{s}}, \end{array}$$

where *β*_*ijw*_^*s*^ means the marginal evacuation time of the dynamic route between *i* and *j* under the traffic condition determined by *F*, that is to say, *β*_*ijw*_^*s*^ is the absolute amount that the value of objective function increases when *F*_*ijw*_^*s*^ increases one unit.

By substituting formulas (35) and (36) into (33), we can obtain

(37) $$\begin{array}{c} \frac{\partial L\left(F,\theta \right)}{\partial F_{ijw}^{s}}=\beta_{ijw}^{s}\left(F\right)-\theta_{ij}. \end{array}$$

Thus formula (31) that describes the Kuhn-Tucker conditions can be written briefly as follows:

(38) $$\begin{array}{c} F_{mnr}^{l}\left(\beta_{mnr}^{l}\left(F\right)-\theta_{mn}\right)=0\;\forall m,n,r,l,\\ \;\left(\beta_{mnr}^{l}\left(F\right)-\theta_{mn}\right)\geq 0\;\forall m,n,r,l. \end{array}$$

Formula (38) shows that the marginal evacuation time of used dynamic routes between each evacuation OD is same at the extreme points, where the arrangements for the departure time and evacuation route are the best. As to the unused dynamic routes, their marginal evacuation time is greater than or equal to that of the used. Thus we conclude that the Lagrange multiplier *θ*_*mn*_ is the minimum evacuation time of all the dynamic routes between *m* and *n*.

### 5.2. Heuristic Algorithm

#### 5.2.1. Framework and Process of This Algorithm

**Figure 1:**
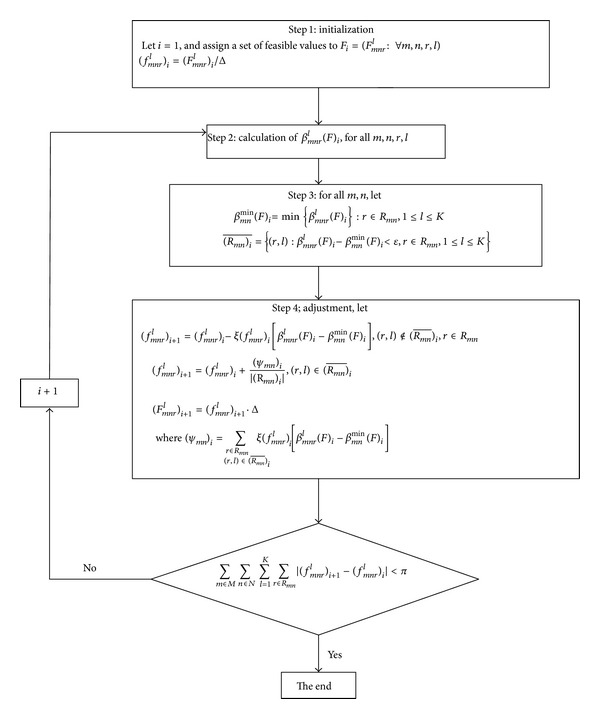
Framework of the algorithm.

In this part, we propose a heuristic algorithm. Once given a set of initial values of the decision variables, the optimal solution can be approximately calculated through continuous iteration and adjustment. Adjustment process is mainly based on the marginal evacuation time, and the goal of the adjustment is to make the marginal evacuation time of used dynamic routes between each evacuation OD equal to the minimum, as well as to make the marginal evacuation time of unused dynamic routes greater than or equal to the minimum marginal evacuation time. The framework and process of the algorithm are shown in Figure 1. The algorithm will not come to an end until it meets certain convergence criteria.

**Step 1**

Give the decision variables a set of initial values. In order to guarantee the feasibility of the initial solution, let

(39) $$\begin{array}{c} {\left(F_{mnr}^{l}\right)}_{1}=\frac{D_{mn}}{\left(J\cdot \Delta \cdot \left|R_{mn}\right|\right)},\;l=1,2,...,J,\\ {\left(F_{mnr}^{l}\right)}_{1}=0,\;l=J+1,J+2,...,K. \end{array}$$

This practice actually distributes the total demand of each evacuation OD to the available dynamic routes evenly, thus ensuring the conservation of the total demand. By choosing an appropriate *J*, we can ensure that all evacuation vehicles will reach the ending node before period *K*. In addition, in order to calculate conveniently, we convert the traffic volume (or the number of vehicles) *F*_*mnr*_^*l*^ on the dynamic route into departure rate *f*_*mnr*_^*l*^.

**Step 2**

Calculate marginal evacuation time of the dynamic routes, and the process will be described in detail in Section 5.2.2.

*Step  3-Step  4*. Adjust current solution according to the marginal evacuation time. For each evacuation OD, comparing the marginal evacuation time of each dynamic route and the minimum marginal evacuation time, if the difference between the two is quite great, then reduce the departure rate of the dynamic route appropriately. If the difference between the two is relatively small, then increase the departure rate. In order to ensure the feasibility of the adjusted solution, the reduction should be evenly allocated to the dynamic routes that need to increase the departure rates.

**Step 5**

Judge the convergence: when the difference between the two decision variable values in adjacent iterations is small enough, the algorithm stops.

Step 2–Step 5 are the process of loop iteration.

As we know, the parameter selected plays an important role in the performance of an algorithm. Here we chose appropriate parameter values through numerous trials. And experiments show that the algorithm can surely and rapidly get global optimum solution and greatly increase the accuracy.

In the case analysis shown in Section 6, in two different evacuation demands, we can get the optimum solutions after iterating 60 times and 21 times, respectively, and compared to other algorithms, there is a big promotion in the convergence rate.

#### 5.2.2. Calculation of Marginal Evacuation Time

*(1) Derivation of Marginal Evacuation Time.* The calculation of marginal evacuation time is the key step in the algorithm, which is also a bridge between the decision variables and adjusting bases. For the dynamic route (*w*, *s*) between certain evacuation OD (*i*, *j*), the marginal evacuation time can be calculated by the following formula which is based on formulas (2) and (36):

(40) βijws(F)=(ls+α·Δ)+tijws(F) +∑m∈M ∑n∈N ∑r∈Rmn ∑l=1KFmnrl·∂∂Fijws(tmnrl(F)).

The three terms on the right of formula (40), respectively, stand for the three aspects of the absolute amount that the total evacuation time increases when the number of vehicles increases one unit. The first term means the waiting time of the new coming vehicle at evacuation source. The second term represents the running time of the new coming vehicle on the route. The third term stands for the effect exerted by the new coming vehicle on other vehicles' running time. For simplicity, assume that the new coming vehicle has no effect on vehicles in different batches, and we only take the effect on vehicles in same batch into consideration. Thus formula (40) can be written briefly as follows:

(41) $$\begin{array}{c} \beta_{ijw}^{s}\left(F\right)=\left(l_{s}+\alpha \cdot \Delta \right)+t_{ijw}^{s}\left(F\right)+F_{ijw}^{s}\frac{\partial }{\partial F_{ijw}^{s}}\left(t_{ijw}^{s}\left(F\right)\right), \end{array}$$

where *l*_*s*_ + *α* · Δ is marginal waiting time and the sum of the last two terms is marginal running time. The value of *t*_*ijw*_^*s*^(*F*) is related to the traffic condition of the road network in evacuation period, and *F*_*ijw*_^*s*^ is determined by the evacuation organization planning, so there exists no analytic function relationship between *t*_*ijw*_^*s*^(*F*) and *F*_*ijw*_^*s*^. It is impossible to calculate the marginal running time of certain dynamic route directly, and we can only calculate it approximately by accumulating the marginal running time of the sections contained in the route.

Assume that route *r* consists of sections *a*_1_, *a*_2_, ..., *a*_*m*_, *β*_*mnr*_^*l*^(*F*) can be calculated by the following formula:

(42) $$\begin{array}{c} \beta_{mnr}^{l}\left(F\right)=t_{1}+\beta_{a_{1}}\left(t_{1}\right)+\beta_{a_{2}}\left(t_{2}\right)+\cdots +\beta_{a_{m}}\left(t_{m}\right), \end{array}$$

where *β*_*a*_*i*__(*t*_*i*_) means the marginal running time of section *a*_*i*_, namely the absolute amount that the total running time increases when the number of vehicles in section *a*_*i*_ increases one unit at time *t*_*i*_. *t*_1_ = *l*_*s*_ + *α* · Δ is the departure time of the new coming vehicle that departs from *m* and *n* along route *r* in period *l*, and it is also the time when the vehicle enters section *a*_1_, so *t*_*i*_, *i* = 2,3, ..., *m*, is the time when the vehicle enters section *a*_*i*_. Moreover, *t*_*i*_ depends on the running and propagation of the corresponding evacuation traffic flow in the road network, and *β*_*a*_*i*__(*t*_*i*_) is determined by the objective function and evacuation traffic flow propagation. So both of the two variables are related to the description method of evacuation traffic flow propagation mechanism.

The running and propagation of the traffic flow in the model are described by a simulation module illustrated in Section 4. The calculation of marginal running time can be carried out in two steps. Firstly, load the corresponding traffic flow into the evacuation network by the simulation module to get the dynamic state of sections in evacuation period that includes the number of vehicles in the sections, inflows and outflows. Then calculate the marginal evacuation time of the dynamic route by accumulating the marginal running time of the sections contained in the route based on formula (42).

*(2) Calculation of Marginal Running Time.* The dynamic marginal running time of a section can be divided into two parts: the marginal running time in the running part and the marginal delay in the queuing part. The marginal running time in the running part means the absolute amount that the total running time increases when the number of vehicles in the section increase one unit at time *t*, namely, the running time of the new coming vehicle in the section according to the above assumptions, as shown in what follows:

(43) $$\begin{array}{c} \beta_{a_{i}}\left(t_{i}\right)=t_{a_{i}}\left(t_{i}\right). \end{array}$$

The marginal delay in the queuing part, which includes the delay of the new coming vehicle itself and other vehicles effected by the former, should be respectively calculated according to different turning directions. Here we only take the effects on vehicles in the same queue into consideration, and the marginal delay can be calculated by following formula:

(44) $$\begin{array}{c} \beta_{a_{i}a_{i+1}}\left(\tau_{i}\right)=\frac{nq_{a_{i}a_{i+1}}\left(\tau_{i}\right)}{yq_{a_{i}a_{i+1}}\left(\tau_{i}\right)}+\frac{1}{yq_{a_{i}a_{i+1}}\left(\tau_{i}\right)}Cnq_{a_{i}a_{i+1}}\left(\tau_{i}\right), \end{array}$$

where *β*_*a*_*i*_*a*_*i*+1__(*τ*_*i*_) is the marginal delay of vehicles that enter the turning queue from section *a*_*i*_ to section *a*_*i*+1_ at time *τ*_*i*_. *C* *nq*_*a*_*i*_*a*_*i*+1__(*τ*_*i*_) represents the number of vehicles in the turning queue from section *a*_*i*_ to section *a*_*i*+1_ influenced by the new coming vehicle at time *τ*_*i*_. Thus, *C* *nq*_*a*_*i*_*a*_*i*+1__(*τ*_*i*_) can be calculated by the following formula:

(45) Cnqaiai+1(τi) =xqaiai+1(τi+nqaiai+1(τi)2yqaiai+1(τi))·nqaiai+1(τi)yqaiai+1(τi).

The marginal evacuation time of route *r*, which consists of sections *a*_1_, *a*_2_,…, *a*_*m*_, can be calculated as follows:

(46) βmnrl(F)=t1+βa1(t1)+βa1a2(τ2)+βa2(t2) +βa2a3(τ2)+⋯+βam−1am(τm−1)+βam(tm),

where *t*_*i*_ and *τ*_*i*_  (*i* = 2,3,…, *m* − 1), respectively, represent the time when the new coming vehicle that departs along route *r* in period *l* enters section *a*_*i*_, and the time when this vehicle enters the queuing part after leaving section *a*_*i*_. The two time variables can be measured by formula (47):

(47) $$\begin{array}{c} \tau_{i}=t_{i}+t_{a_{i}}\left(t_{i}\right),\\ t_{i+1}=\tau_{i}+\frac{nq_{a_{i}a_{i+1}}\left(\tau_{i}\right)}{yq_{a_{i}a_{i+1}}\left(\tau_{i}\right)}\\ i=2,3,\ldots ,m-1, \end{array}$$

where *nq*_*a*_*i*_*a*_*i*+1__(*τ*_*i*_)/*yq*_*a*_*i*_*a*_*i*+1__(*τ*_*i*_) is used to calculate the delay of the vehicles that enter the turning queue from section *a*_*i*_ to section *a*_*i*+1_ after finishing section *a*_*i*_ at time *τ*_*i*_.

## 6. Case Analysis

**Figure 2:**
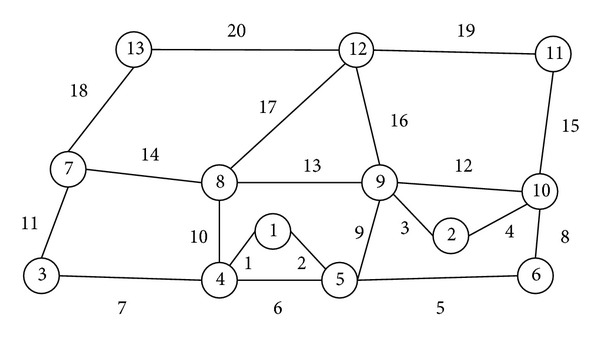
Typical evacuation network.

In this section, we will analyze the evacuation in areas of Beijing. The evacuation routes in Beijing have the following typical characteristics: on the whole, all of the evacuation routes look like a checkerboard, and generally there are crisscrossed small road networks around the evacuation sources. In this way, we can draw a typical evacuation road network as shown in Figure 2.

The basic parameters setting, traffic capacity at turning intersections, and available routes between each evacuation OD are, respectively, shown in Tables 1, 2, and 3. For the sake of simplicity, let *t* = 6, ∆ = 6 (min), *ξ* = 0.01, *ε* = 1.98, and convergence parameter *π* = 1.532.

By the aforementioned model and algorithm, we can get the optimal evacuation organization planning (shown in Tables 4 and 5) corresponding to two different evacuation demands after iterating 60 times and 21 times, and the parameters of the two demands are (*D*_1,13_ = 1000, *D*_2,13_ = 1500), (*D*_1,13_ = 300, *D*_2,13_ = 500). Moreover, the number of vehicles that depart along different routes are shown in the tables and the respective evacuation time is 59 minutes and 25 minutes.

## 7. Conclusions

Due to the basic features of major incident and characteristics in urban area, once a major incident occurs, it subsequently leads to great losses. Therefore, the government needs to improve its ability for emergency management. This paper establishes a comprehensive model for the optimization of emergency evacuation route and departure time based on the simulation. In the process, we find reasonable description method of evacuation traffic flow propagation under traffic control, and also propose a heuristic algorithm for the optimization of this comprehensive model. In case analysis, we take some areas in Beijing as evaluation sources to verify the reliability of this model. All in all, our evaluation model can be applied to the actual situation under traffic control.

## Figures and Tables

**Table 1:** Basic parameters setting.

Section	Running speed	Section length	Traffic capacity
	*V* (km/h)	*L* (m)	*C* (c/h)
(1–4)	10	600	1900
(1–5)	10	600	1100
(2–9)	15	600	1200
(2–10)	13	600	2100
(6-5)	15	800	1900
(5-4)	14	800	1850
(4-3)	16	800	2000
(10–6)	9	900	1700
(5–9)	13	900	1100
(4–8)	16	900	2200
(3–7)	10	900	2300
(10-9)	10	850	1850
(9-8)	15	850	1950
(8-7)	13	900	2100
(10-11)	12	1200	1200
(9–12)	14	1000	2500
(8–12)	12	1000	1800
(7–13)	13	900	1050
(11-12)	15	900	1970
(12-13)	10	800	2050

**Table 2:** Traffic capacity at turning intersections.

Intersection	Turning direction	Traffic capacity	Intersection	Turning direction	Traffic capacity
	Node	(c/h)		Node	(c/h)
3	(4-3-7)	1800		(10-9-8)	1000
4	(1-4-3)	960	9	(10-9-12)	1100
	(1-4-8)	1060		(2-9-8)	1200
	(5-4-3)	940		(2-9-12)	960
	(5-4-8)	1130		(2-10-9)	540
5	(1-5-4)	600	10	(2-10-11)	720
	(6-5-4)	570		(6-10-9)	1120
6	(10-6-5)	1100		(6-10-11)	1200
7	(3-7-13)	1220	11	(10-11-12)	1400
	(8-7-13)	1230		(8-12-13)	1200
8	(4-8-7)	560	12	(9-12-13)	1050
	(4-8-12)	680		(11-12-13)	1350
	(9-8-7)	720			
	(9-8-12)	800			

**Table 3:** Available routes between each evacuation OD.

OD	Route number	Route (a sequence of nodes)
1-13	1	1-4-3-7-13
	2	1-4-8-12-13
	3	1-4-8-7-13
	4	1-5-4-3-7-13
	5	1-5-4-8-12-13
	6	1-5-4-8-7-13
	7	1-5-9-8-7-13
	8	1-5-9-8-12-13
	9	1-5-9-12-13

2-13	10	2-9-8-7-13
	11	2-9-8-12-13
	12	2-9-12-13
	13	2-10-9-8-7-13
	14	2-10-9-8-12-13
	15	2-10-9-12-13
	16	2-10-11-12-13
	17	2-10-6-5-4-3-7-13
	18	2-10-6-5-4-8-12-13
	19	2-10-6-5-4-8-7-13
	20	2-10-6-5-4-3-7-13
	21	2-10-6-5-5-4-8-12-13
	22	2-10-6-5-5-4-8-7-13
	23	2-10-6-5-5-9-8-7-13
	24	2-10-6-5-5-9-8-12-13
	25	2-10-6-5-5-9-12-13

**Table 4:** Optimal evacuation planning (*D*_1,13_ = 1000, *D*_2,13_ = 1500).

Departure time (every 6 minutes)									
OD	Route	1	2	3	4	5	6	7	8
1-13	1	59	66	83	96				
	2	40	58	51	50				
	3	22	32	37	37				
	4	22	34	32	8				
	5	7	7	6	2				
	6	8	12	12	3				
	7	12	18	16	4				
	8	8	35	39	11				
	9	20	20	23	18				

2-13	10	40	53	75	50	15	19	12	6
	11	—	10	7	17	12	6		
	12	16	17	15	21	13	8	2	
	13	8	11	12	14	5	6		
	14	31	18	37	44	8			
	15	12	15	16	17	3	5		
	16	8	14	12	7				
	17	30	11	30	25	22	6		
	18	5	55	68	73				
	19	14	8	12					
	20	16	8						
	23	4							

**Table 5:** Optimal evacuation planning (*D*_1,13_ = 300, *D*_2,13_ = 500).

Departure time (every 6 minutes)				
OD	Route	1	2	3
1-13	1	80	29	9
	2	52	16	3
	3	20	13	6
	4	10	7	3
	5	8	6	5
	6	16	5	7
	8	13	4	5
	9	10	7	2

2-13	10	80	50	5
	11	6	3	1
	12	6	6	—
	13	30	20	6
	14	35	7	2
	15	72	45	13
	16	15	4	5

## References

[1] Desmet A, Gelenbe E (2013). Graph and analytical models for emergency evacuation. *Future Internet*.

[2] Caunhye AM, Nie X, Pokharel S (2012). Optimization models in emergency logistics: a literature review. *Socio-Economic Planning Sciences*.

[3] Pel AJ, Bliemer MCJ, Hoogendoorn SP (2012). A review on travel behaviour modelling in dynamic traffic simulation models for evacuations. *Transportation*.

[4] Chen YM, Xiao DY (2008). Emergency evacuation model and algorithms. *Journal of Transportation Systems Engineering and Information Technology*.

[5] Kwon E, Pitt S (2005). Evaluation of emergency evacuation strategies for downtown event traffic using a dynamic network model. *Transportation Research Record*.

[6] Cova TJ, Johnson JP (2003). A network flow model for lane-based evacuation routing. *Transportation Research A*.

[7] Petruccelli U (2003). Urban evacuation in seismic emergency conditions. *ITE Journal*.

[8] Zawack DJ, Thompson GL (1987). A dynamic space-time network flow model for city traffic congestion. *Transportation Science*.

[9] Yang B, Wu YG, Ren B (2012). Application of multi-resolution modelling in emergency evacuation simulation. *Simulation and Process Modeling*.

[10] Pelechano N, Malkawi A (2008). Evacuation simulation models: challenges in modeling high rise building evacuation with cellular automata approaches. *Automation in Construction*.

[11] Jha M, Moore K, Pashaie B (2004). Emergency evacuation planning with microscopic traffic simulation. *Transportation Research Record*.

[12] Chiu YC, Zheng H, Villalobos J, Gautam B (2007). Modeling no-notice mass evacuation using a dynamic traffic flow optimization model. *IIE Transactions*.

[13] Pidd M, De Silva FN, Eglese RW (1996). A simulation model for emergency evacuation. *European Journal of Operational Research*.

[14] Hasby M, Khodra ML Optimal path finding based on traffic information extraction from Twitter.

[15] Wu L, Lin H A case study of developing personalized spatial cognitive road network and raster capable route finding algorithm for pedestrian evacuation behavior simulation.

[16] Li D, Zhang X, Wang L (2013). On the crowded places multi-exits emergency evacuation model and algorithm. *International Journal of Computer Science Issues*.

[17] Lu Q, George B, Shekhar S (2005). Capacity constrained routing algorithms for evacuation planning: a summary of results. *Advances in Spatial and Temporal Databases*.

[18] Sherali HD, Carter TB, Hobeika AG (1991). A location-allocation model and algorithm for evacuation planning under hurricane/flood conditions. *Transportation Research Part B*.

[19] Papinigisa V, Gedabcand E, Lukošius K (2010). Design of people evacuation from rooms and buildings. *Journal of Civil Engineering and Management*.

[20] Dunn CE, Newton D (1992). Optimal routes in GIS and emergency planning applications. *Area*.

[21] Yamada T (1996). A network flow approach to a city emergency evacuation planning. *International Journal of Systems Science*.

[22] Campos VBG, da Silva PAL, Netto POB Evacuation transportation planning: a method of identify optimal independent routes.

[23] Mamada S, Makino K, Fujishige S The evacuation problem, dynamic network flows, and algorithms.

